# The interaction of enteric bacterial effectors with the host engulfment pathway control innate immune responses

**DOI:** 10.1080/19490976.2021.1991776

**Published:** 2021-11-01

**Authors:** Ibrahim M Sayed, Stella-Rita Ibeawuchi, Dominique Lie, Mahitha Shree Anandachar, Rama Pranadinata, Manuela Raffatellu, Soumita Das

**Affiliations:** a Department of Pathology, University of California San Diego, La Jolla, CA, USA; b Department of Pediatrics, Division of Host-Microbe Systems and Therapeutics, University of California San Diego, LA Jolla, CA, USA; c Center for Mucosal Immunology, Chiba University-UC San Diego, La Jolla, CA USA

**Keywords:** ELMO1, bacterial effectors, Wxxxe motif, commensals, innate immune response

## Abstract

Host engulfment protein ELMO1 generates intestinal inflammation following internalization of enteric bacteria. In *Shigella*, bacterial effector IpgB1 interacts with ELMO1 and promotes bacterial invasion. IpgB1 belongs to the WxxxE effector family, a motif found in several effectors of enteric pathogens. Here, we have studied the role of WxxxE effectors, with emphasis on *Salmonella* SifA and whether it interacts with ELMO1 to regulate inflammation. In-silico-analysis of WxxxE effectors was performed using BLAST search and Clustal W program. The interaction of ELMO1 with SifA was assessed by GST pulldown assay and co-immunoprecipitation. ELMO1 knockout mice, and ELMO1-depleted murine macrophage J774 cell lines were challenged with WT and *SifA* mutant *Salmonella*. Bacterial effectors containing the WxxxE motif were transfected in WT and ELMO1-depleted J774 cells to assess the inflammatory cytokines. ELMO1 generates differential pro-inflammatory cytokines between pathogenic and nonpathogenic bacteria. WxxxE motif is present in pathogens and in the TIR domain of host proteins. The C-terminal part of ELMO1 interacts with SifA where WxxxE motif is important for interaction. ELMO1–SifA interaction affects bacterial colonization, dissemination, and inflammatory cytokines *in vivo*. Moreover, ELMO1–SifA interaction increases TNF-α and IL-6 production from the macrophage cell line and is associated with enhanced Rac1 activity. ELMO1 also interacts with WxxxE effectors IpgB1, IpgB2, and Map and induces inflammation after challenge with microbes or microbial ligands. ELMO1 generates a differential response through interaction with the WxxxE motif, which is absent in commensals. ELMO1-WxxxE interaction plays a role in bacterial pathogenesis and induction of inflammatory response.

## Introduction

Host defense detects the presence of harmful bacteria to initiate a protective response. The host immune cells recognize the pathogen-associated molecular patterns (PAMPs) of bacteria through their pattern recognition receptors (PRRs).^1–6^ Although lipopolysaccharide (LPS) is a key cell wall component of both pathogenic and commensal Gram-negative bacteria, host defenses exhibit a differential immune response against bacteria that affect the ability to cause disease.^7,8^ The mechanisms by which host sensors of microbes can differentiate between pathogens and commensals are not completely recognized.

Previously, we showed that the host engulfment protein called EnguLfment and cell MOtility protein 1 (ELMO1) plays a crucial role in the internalization of enteric pathogens, regulation of autophagy induction, and bacterial clearance during enteric infection.^9,10^ ELMO1 is a cytosolic protein that interacts with another PRR called Brain Angiogenesis Inhibitor 1 (BAI1) which binds with the oligosaccharide core of the LPS of Gram-negative bacteria.^11,12^ ELMO1 also interacts with the cytosolic protein Dock180, and they together act as a guanine nucleotide exchange factor for the small Rho GTPase Ras-related C3 botulinum toxin substrate 1 (Rac1), leading to actin cytoskeleton reorganization and bacterial engulfment.^9,11^ The polymorphism of ELMO1 is involved in several inflammatory diseases, such as inflammatory bowel disease, rheumatoid arthritis, kidney disease, and diabetic nephropathy.^13–15^ Recently, we showed that ELMO1 expression is elevated in the colonic epithelium of IBD patients, where higher expression is positively correlated with the elevated expression of pro-inflammatory cytokines, MCP-1, and TNF-α.^16^ However, whether ELMO1 differentially initiates the immune response after sensing pathogens and/or commensals remains to be determined.

To survive and proliferate inside the host, pathogens utilize a variety of secretion systems (types I–VI) that target host proteins to hijack host defense mechanisms.^17–19^ Some bacterial effectors target the host cytoskeleton G protein signaling cascades of the Rho family of GTPases resulting in induction of cytoskeletal rearrangements and facilitating the bacterial entry.^20,21^ Previously it has been shown that ELMO1 interacts with IpgB1, a WxxxE effector of *Shigella* and controls internalization in epithelial cells.^22^ Similar to IpgB1 and IpgB2 of *Shigella*, other bacterial effectors, such as Map from enteropathogenic *Escherichia coli* (EPEC) and SifA from *Salmonella*, bypass the endogenous RhoGTPases to directly activate downstream signaling responses.^17^ These effectors have a conserved sequence similarity in their C-terminal targeting sequences entitled “WxxxE motif.”^17^ The WxxxE motif is reported in 24 effector proteins present in enteric pathogens, and they can all utilize a similar molecular mechanism.^17^ However, whether the WxxxE motif is unique to pathogens, and if so whether it could promote an immune response mediated by ELMO1 that discriminates between pathogens and commensals, is not known.

Here, we investigated whether ELMO1 interacts with other WxxxE effectors and how the effector–ELMO1 interaction controls inflammatory responses. Our in-silico analysis identified WxxxE signature motif to be predominantly present in enteric pathogens, plant pathogens, and in the Toll/interleukin-1 receptor (TIR) homology domain of both host and pathogens but absent in commensals. Using *Salmonella* as a model for enteric pathogens, we found that the WxxxE motif is important to maintain the interaction between the *Salmonella* SifA effector and ELMO1. ELMO1-KO mice and ELMO1-depleted macrophages were used to study the impact of ELMO1–SifA interaction on bacterial colonization, dissemination, and inflammatory response. ELMO1 also interacts with WxxxE effectors from *Shigella* and *E. coli* and the interaction of ELMO1 with WxxxE effectors induces the production of pro-inflammatory cytokines.

## Results

### ELMO1 generates a differential immune response between pathogenic and nonpathogenic bacteria

**Figure 1.**
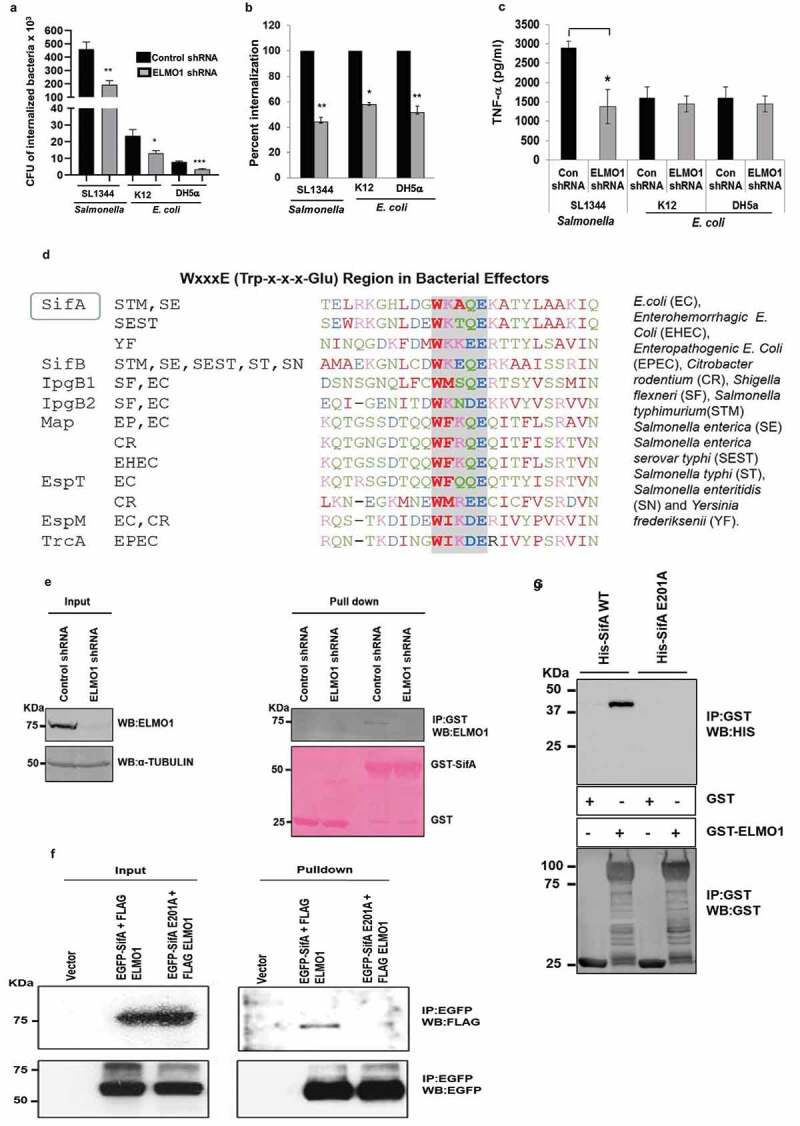
**ELMO1 generates a differential immune response between pathogenic and nonpathogenic bacteria**. (a) Bacterial internalization was measured in control and ELMO1 shRNA J774 cells challenged with *Salmonella enterica* serovar Typhimurium (SL1344), *E. coli* strain K12, and *E. coli* DH5α. (b) The percentage of bacterial internalization was compared between control and ELMO1 shRNA J774 cells, bacterial internalization for control cells was taken as 100% as performed in A. (c) The level of TNF-α was measured in control and ELMO1 shRNA J774 cells challenged with SL1344, *E. coli* K12, and *E. coli* DH5α after 3 h. Data in (a), (b), and (c) represent the mean ± SEM of three independent experiments. *, **, *** means *p* ≤ 0.05, ≤ 0.01, and ≤ 0.001, respectively, as assessed by unpaired two-tailed Student t-test. (d) BLAST-Protein search using the amino acid sequence of *Shigella* IpgB1 with all other bacteria identified sequence similarities with a signature motif (WxxxE or Trp-x-x-x-Glu) present in bacterial effectors from enteric pathogens but absent in commensals. (e) GST pulldown was performed with control GST and GST-SifA with the lysates from control and ELMO1 shRNA J774 cells. The input was shown on the left side and immunoblotted with anti-ELMO1 antibody. α -Tubulin was used as a loading control. (f) HEK 293 cells were transfected with either the vector control or with the EGFP-SifA (with WxxxE signature motif)+FLAG-ELMO1 or with the EGFP-SifAE201A (WxxxE mutant motif)+FLAG-ELMO1. The lysates from each condition were used either as input (left) or used for EGFP pulldown, followed by immunoblotting with anti-FLAG antibody to know the level of FLAG-ELMO1. Equal loading is confirmed by the EGFP antibody (lower panel). (g) GST pulldown with either the GST alone or with GST-ELMO1 was incubated with His-SifA WT (with WxxxE signature motif) and His-SifA E201A (WxxxE mutant motif). The pulldown samples were immunoblotted with anti-His antibody (upper panel). The equal loading of beads is confirmed by anti-GST antibody (lower panel)

We have previously shown that ELMO1 is involved in the internalization of *Salmonella*.^9^ To understand the role of ELMO1 in the internalization of pathogenic and nonpathogenic bacteria, we infected control and ELMO1 shRNA J774 macrophages with the pathogen *Salmonella* and with two nonpathogenic *E. coli* strains (K12 and DH5α). Gentamicin protection assay showed that the number of internalized bacteria was low in control shRNA cells after infection with the nonpathogenic *E. coli* strains compared to *Salmonella* (Figure 1(a)). Bacterial internalization was lower in ELMO1 shRNA cells compared to control cells irrespective of pathogenic or nonpathogenic bacteria. The percentage internalization (Figure 1(b)) showed ELMO1-dependent bacterial internalization in all three cases, with a significant ~50% reduction of internalization in ELMO1 shRNA cells compared to control cells.

To assess whether ELMO1 generates a differential immune response between pathogenic and nonpathogenic bacteria, we measured the level of pro-inflammatory cytokine TNF-α released from control and ELMO1 shRNA cells. TNF-α levels were significantly lower in ELMO1 shRNA cells compared to control cells infected with *Salmonella*. However, the level of TNF-α was comparable in control and ELMO1 shRNA cells infected with nonpathogenic *E. coli* (Figure 1(c)). This finding suggests that this ELMO1-dependent cytokine response is likely triggered by a virulence factor from *Salmonella*. Previously, we have shown that the TNF-α response in macrophages after interaction with microbial ligands (LPS and lipoteichoic acid) does not depend on ELMO1.^9^ While searching for bacterial factors potentially involved in the ELMO1-dependent differential cytokine responses, we found a previous report where ELMO1 interacts with the *Shigella* effector IpgB1.^22^ Of note, IpgB1 belongs to the WxxxE effector groups and shares the signature motif WxxxE (Tryptophan-xxx-Glutamate) found only in enteric pathogens.^17^ A BLAST database search identified the conserved signature WxxxE motif as widely distributed among enteric pathogens (Figure 1(d)**, Supplementary** Figure 1(a)), non-enteric pathogens including *Acinetobacter* and *Klebsiella* (**Supplementary** Figure 1(b)), and plant pathogens (**Supplementary** Figure 1(c)), but not among commensals. Interestingly, the WxxxE motif is also present in the TIR domain of a subset of host TLRs (TLR 1, 4, 6, 7, 8, 9, 10) and TLR1, 4, 6, 10 have 2 WxxxE motifs (**Supplementary** Figure 1(d)).

Based on the bioinformatics analysis that identified the WxxxE motif in the *Salmonella* effector SifA (Figure 1(d)), we examined whether *Salmonella* SifA interacts with endogenous ELMO1 in macrophages (Figure 1(e)**, Supplementary** Figure 1(e)). GST pulldown assay showed ELMO1 in J774 control shRNA cells and primary bone marrow-derived macrophages (BMDM) bound to GST-SifA and the specificity was further confirmed when J774 ELMO1 shRNA cells failed to bind the GST-SifA. To further test the impact of the WxxxE signature motif, we performed immunoprecipitation with FLAG-tagged-ELMO1 in cells co-transfected with either EGFP-SifA wild type or EGFP-SifA-E201A mutant. We chose the mutation of the glutamine (E) but not the tryptophan (W) residue because a previous study showed that a mutation in W of the WxxxE motif affects the stability and/or tertiary structure of SifA and impedes its interaction with SKIP.^23^ Mutation of the WxxxE_201_ motif abolished the interaction between SifA and ELMO1 when we performed the immunopulldown (IP) with EGFP-conjugated beads (figure 1(f)). We further confirmed the specificity of the interaction by GST pulldown with purified His-tagged SifA WT or E201A mutant and GST ELMO1 (Figure 1(g)). The results indicate the WxxxE motif is important for the interaction of SifA with ELMO1.

### *ELMO1–SifA interaction affects bacterial dissemination and inflammatory responses* in vivo

**Figure 2.**
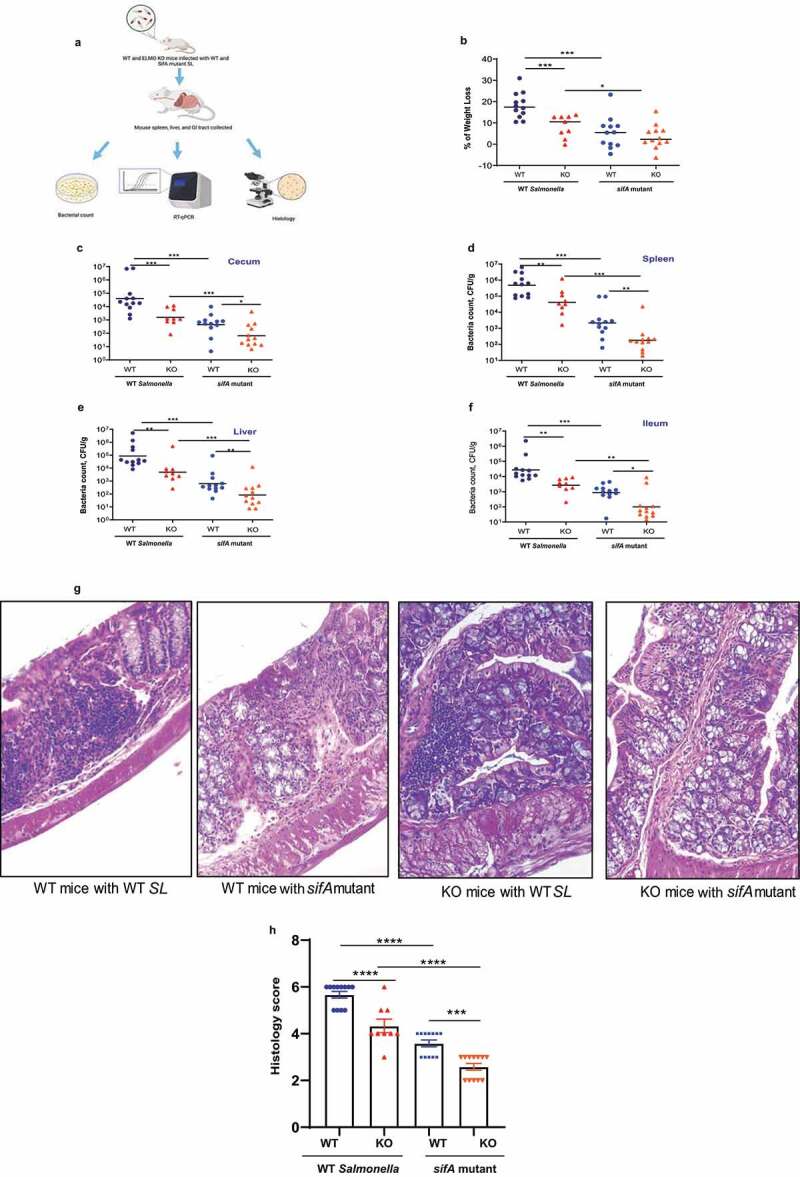
**Infection of WT and global ELMO1KO mice with WT *Salmonella* (*SL*) and *sifA* mutant shows the involvement of ELMO1–SifA interaction in bacterial dissemination and inflammatory responses**. (a) Schematic diagram represents the experimental design. (b) The percentage of weight loss was measured in WT and global ELMO1 KO mice infected via oral gavage with WT *SL* and *sifA* mutant strains for 5 days. (c-f) Bacterial burden was assessed at day 5 of infection in the cecum (c), spleen (d), liver (e), and ileum (f) of WT and global ELMO1 KO mice infected with WT *SL* and *sifA* mutant strains. (g-h) The H&E staining (g) and the pathology score (h) were assessed based on the degree of crypts loss, the infiltration of leukocytes in both mucosa and submucosa of WT and global ELMO1 KO mice infected with WT *SL* and *sifA* mutant. *, **, ***, and **** mean *p* ≤ 0.05, ≤ 0.01, ≤ 0.001, and ≤ 0.0001, respectively as assessed by Mann Whitney test

By using ELMO1-knockout (KO) mouse, we have previously shown that ELMO1 promotes *Salmonella* dissemination and intestinal inflammation.^9^ To assess the relevance of ELMO1 and SifA interaction on bacterial gut colonization, dissemination, and inflammatory responses *in vivo*, WT and global ELMO1 KO mice were infected with *Salmonella enterica* serovar Typhimurium wild-type strain SL1344 (WT *SL*) or with an isogenic *sifA* mutant with a dose of 5 × 10^7^ CFU/mouse for 5 days by oral gavage. Samples from the ileum, cecum, spleen, and liver were collected to measure bacterial burden and the level of inflammatory cytokines as shown in the schematics in Figure 2(a).

Five days after infection, the percentage of weight loss was significantly higher in WT mice compared to ELMO1 KO mice infected with WT *SL*, and higher weight loss was recorded in mice infected with WT *SL* compared to the littermates infected with the *sifA* mutant (Figure 2(b)). The bacterial burden in the cecum, spleen, liver, and ileum (Figures 2(c)**-2 F)** was lower in mice infected with the *sifA* mutant compared to mice infected with WT *SL*, with a significant decrease in the bacterial load in ELMO1 KO mice compared with WT mice (Figures 2(c)**-2 F)**. H&E staining demonstrated loss of crypts and dense infiltration of leukocytes in both mucosa and submucosa of the infected mice ileum with a higher inflammatory infiltrate in WT *SL*-infected mice compared to mice infected with the *sifA* mutant (Figure 2(g)). The histology score was significantly higher in WT mice compared to ELMO1 KO mice regardless of the inoculated strain (Figure 2(h)). Overall, the degree of infection and inflammation was lower in mice infected with the *sifA* mutant, and it was the lowest in ELMO1 KO mice infected with the *sifA* mutant.

Since ELMO1 is expressed both in epithelial cells and myeloid cells, we aimed to assess the effect of ELMO1 expression on myeloid cells, its interaction with SifA *in vivo*, and the impact of this interaction on *Salmonella* dissemination. To this end, we orally infected WT and ELMO1 KO in myeloid cell-specific (LysM-cre-driven)^9^ mice with WT *SL* or the *sifA* mutant for 5 days. Similar to the global KO mice, the bacterial burden in the spleen and ileum was lower in mice infected with the *sifA* mutant compared to mice infected with WT *SL*, with a significant decrease in the bacterial load in LysM-cre- ELMO1 KO mice compared with WT mice **(Supplementary** Figure 2). Next, we assessed the relevance of ELMO1 and SifA interaction in the early phase of infection (infection by gavage for 2 days) in WT and global ELMO1 KO mice. In WT mice, WT *SL* infection caused a significant weight loss and higher bacterial burden in the cecum, liver, spleen, but not in the ileum, compared to the *sifA* mutant **(Supplementary** Figure 3 **A-E)**. In contrast, we could not find any difference between WT *SL* and the *sifA* mutant in ELMO1 KO mice **(Supplementary** Figure 3 **A-E)**. The bacterial burden and weight loss were slightly higher in WT mice compared to ELMO1 KO mice in WT *SL* infection **(Supplementary** Figure 3 **A-E)**.

**Figure 3.**
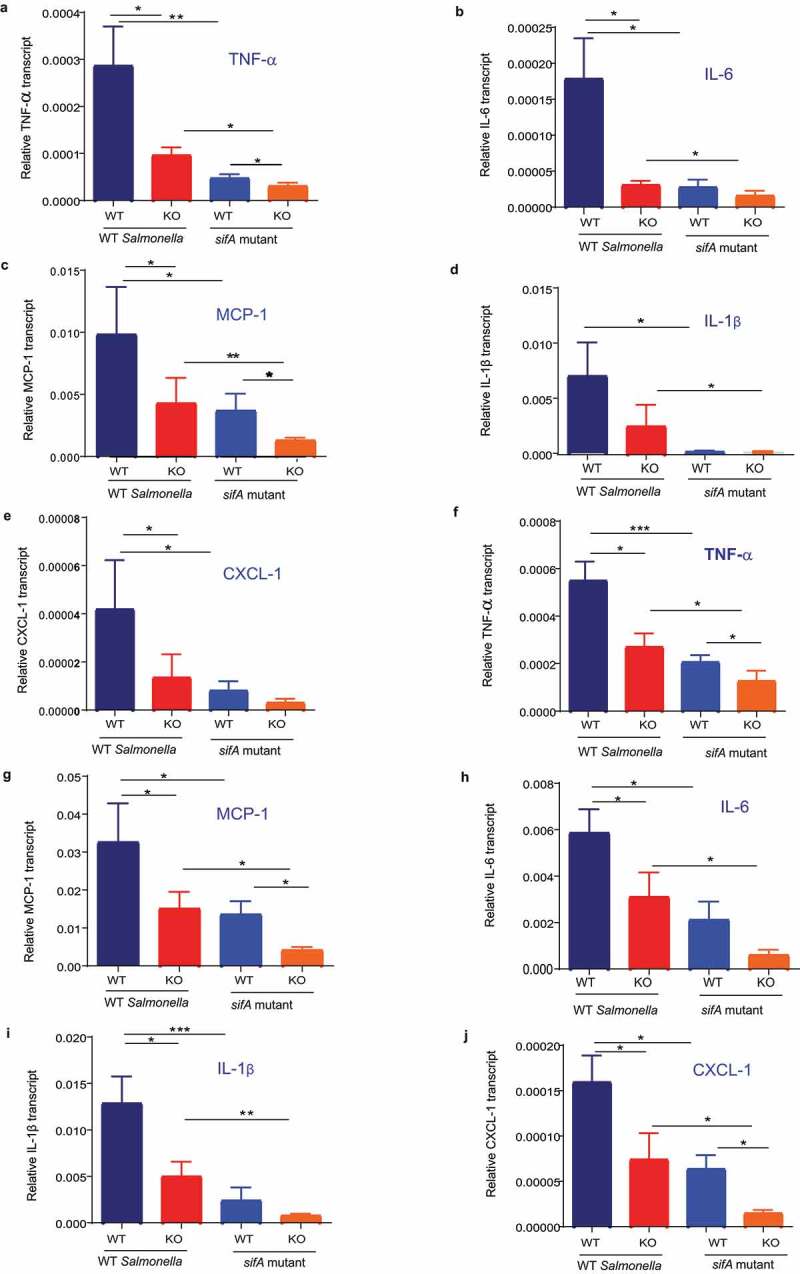
**Infection of WT and global ELMO1 KO mice with WT *SL* and *sifA* mutant shows the involvement of ELMO1–SifA interaction on the induction of innate immune responses**. (a-e) Total RNA was isolated from the cecum of WT and global ELMO1 KO mice infected with WT *SL* and *sifA* mutant for 5 days as in Figure (2), the transcript level of inflammatory cytokines such as TNF-α (a), IL-6 (b), MCP-1 (c), IL-1β (d), and CXCL-1 (e) was measured by RT-qPCR. (f-j) Total RNA was isolated from the spleen of WT and global ELMO1 KO mice infected with WT *SL* and *sifA* mutant in Figure (2), the transcript level of inflammatory cytokines such as TNF-α (f), MCP-1 (g), IL-6 (h), IL-1β (i), and CXCL-1 (j) was measured by RT-qPCR. *, **, *** means *p* ≤ 0.05, ≤ 0.01, and 0.001, respectively as assessed by Mann Whitney test

To assess the role of ELMO1–SifA interaction in regulating inflammatory responses and in the induction of innate immune responses *in vivo*, the expression of inflammatory transcripts, such as TNF-α, MCP-1, IL-6, IL-1β, and CXCL-1 was assessed by RT-qPCR in the cecum (Figure 3 **A-E**) and spleen (Figure 3 **F-J)** of WT mice and ELMO1 KO mice infected with WT *SL* or the *sifA* mutant. The expression of inflammatory cytokines was significantly reduced in mice infected with the *sifA* mutant compared to WT *SL* infected mice, with much decrease in the levels of these transcripts in ELMO1 KO mice compared with the WT mice in both infections. Similar to the bacterial burden and histology score, the expression of inflammatory cytokines had a similar finding where WT mice infected with WT *SL* showed the highest pro-inflammatory cytokines and the ELMO1 KO mice infected with *sifA* mutant had the lowest pro-inflammatory cytokines (Figure 3). In early kinetic of infection (after 2 days of infection), the transcript level of inflammatory cytokines was higher in WT mice infected with WT *SL* compared to the other groups. ELMO1 KO mice infected with the *sifA* mutant showed the lowest level of inflammatory transcripts **(Supplementary** Figure 4).

**Figure 4.**
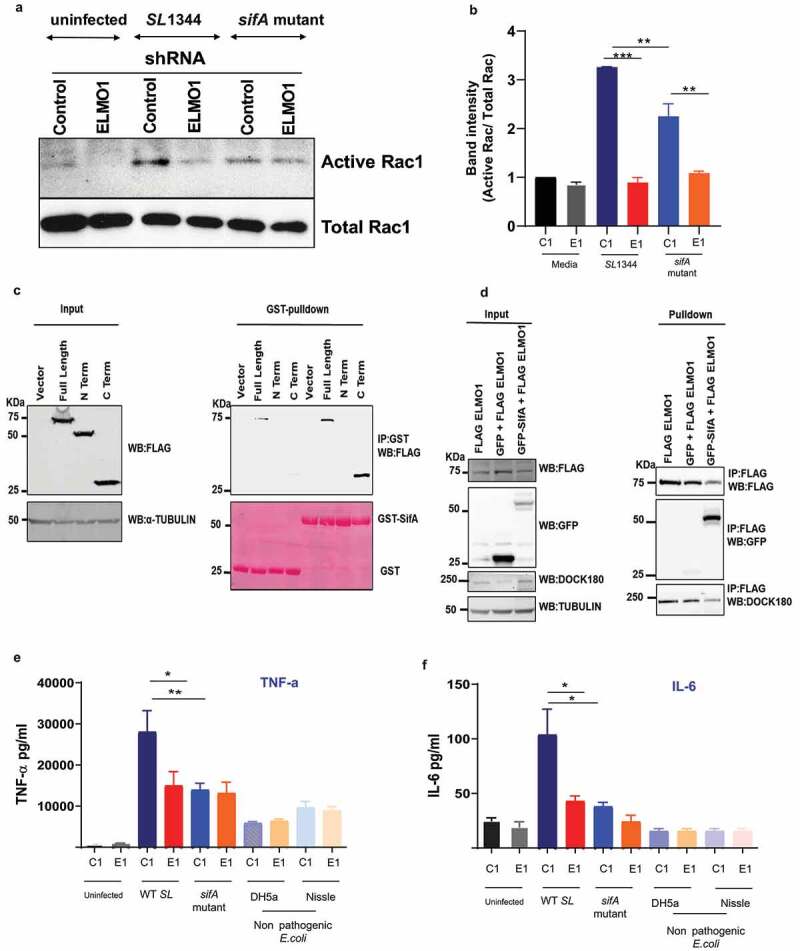
**Effect of ELMO1–SifA interaction on induction of immune responses in control and ELMO1 shRNA cells** (a) Control (C1) or ELMO1 shRNA (E1) J774 cells were infected with WT *SL* or *sifA* mutant strain for 1 h, Rac1 Activity was assessed by pull down assay using GST-PBD beads, followed by WB for active (GTP bound) Rac1 (upper panel) and total Rac1 (lower panel) using Rac1 Antibody. (b) The band intensity for active Rac1 in (A) was normalized to total Rac1 and the ratio of active Rac1/ total Rac1 was quantified in all samples. The densitometry was done with 3 individual experiments **. *** means *p* < .01 and 0.001, respectively. (c) HEK293 cells transfected with FLAG tagged ELMO1-full length (FL) (aa 1-727), FLAG tagged ELMO1- N terminal (NT) (aa-1-532) and FLAG tagged ELMO-1- C terminal (CT) (aa-532-727) , were lysed and incubated with GST-SifA immobilized to glutathione-sepharose affinity beads. . (Right) represents pulldown assays using anti-Flag antibody (upper panel) and Ponceau S stain (lower panel). (Left): Western blot of the input cell lysates using anti-Flag antibody (upper panel), and α-Tubulin was used as a loading control (d) Co-immunoprecipitation assay to check any interference in the binding of Dock180 when ELMO1 interacts with SifA. FLAG-ELMO1 and GFP-SifA were transfected in HEK293 cells followed by cell lysis and IP using anti-FLAG antibody. Proteins were visualized by immunoblotting with corresponding antibodies. (e-f) Control (C1) or ELMO1 shRNA (E1) J774 cells were infected with WT SL1344, *sifA* mutant strain, and nonpathogenic *E. coli* (e), IL-6 (f), was measured by ELISA in the supernatant of infected cells. *, ** means *p* ≤ 0.05, and ≤ 0.01, respectively as assessed by one-way ANOVA multiple comparisons

### *Impact of ELMO1–SifA interaction on the macrophage immune response* in vitro

ELMO1 binds and stabilizes Dock180, which in turn activates Rac1.^24^ Interestingly, we found that ELMO1 activates Rac1 during *Salmonella* infection.^9^ To assess the impact of ELMO1–SifA interaction on Rac1 activation, control and ELMO1 shRNA J774 cells were infected with WT *SL* or the *sifA* mutant strain for 1 h (Figure 4(a)). The amount of active Rac1 was higher after infection with WT *SL* in control shRNA cells compared to ELMO1 shRNA cells. The *sifA* mutant showed a reduction in Rac1 activity compared to the WT *SL* strain. The densitometry in Figure 4(b) confirmed that ELMO1 shRNA cells have the lowest active Rac1, and the level was comparable after infection with WT *SL* or the *sifA* mutant. These results suggest that ELMO1 may interact with other effectors and controls the active Rac1.

Since the N-terminal part of Dock180 interacts with the C-terminal part of ELMO1^25,26^ and controls Rac1 activity, we investigated the interaction of SifA with ELMO1 using the N-terminal and C-terminal part of ELMO1. GST pulldown with GST-SifA showed that SifA is bound by both the full-length ELMO1 and the C-terminal part of ELMO1 but not the N-terminal part of ELMO1 (Figure 4(c)). As Dock180 and SifA both bind to ELMO1 in the C-terminus, we checked whether there is any interference between their interaction. The co-immunoprecipitation of FLAG-ELMO1 and GFP-SifA in HEK293 cells showed that Dock180 can interact with ELMO1 in the presence of SifA (Figure 4(d)).

Next, we assessed the impact of ELMO1–SifA interaction on the immune response generated from the macrophages *in vitro*. We measured the level of inflammatory cytokines, such as TNF-α and IL-6, in control and ELMO1 shRNA J774 cells after infection with WT *Salmonella*, with the *sifA* mutant, or with nonpathogenic bacteria, such as *E. coli* Nissle-probiotic strain and *E. coli* DH5α. As expected, we did not detect any difference in the level of cytokines in the control and ELMO1 depleted cells infected with nonpathogenic bacteria (Figures 4(e)**-4 F)**. Similarly, the level of TNF-α and IL-6 was comparable in control and ELMO1 shRNA cells infected with the *sifA* mutant (Figures 4(e)**-4 F)**. On the other hand, the level of TNF-α and IL-6 was significantly higher in control cells compared to ELMO1 shRNA cells during the infection with WT *SL* (Figures 4(e)**-4 F)**, and the level of these cytokines was significantly higher in control cells infected with WT *SL* compared to control cells infected with the *sifA* mutant (Figures 4(e)**-4 F)**.

### Other enteric bacterial effectors containing WxxxE motif interact with ELMO1 and controls pro-inflammatory cytokines

**Figure 5.**
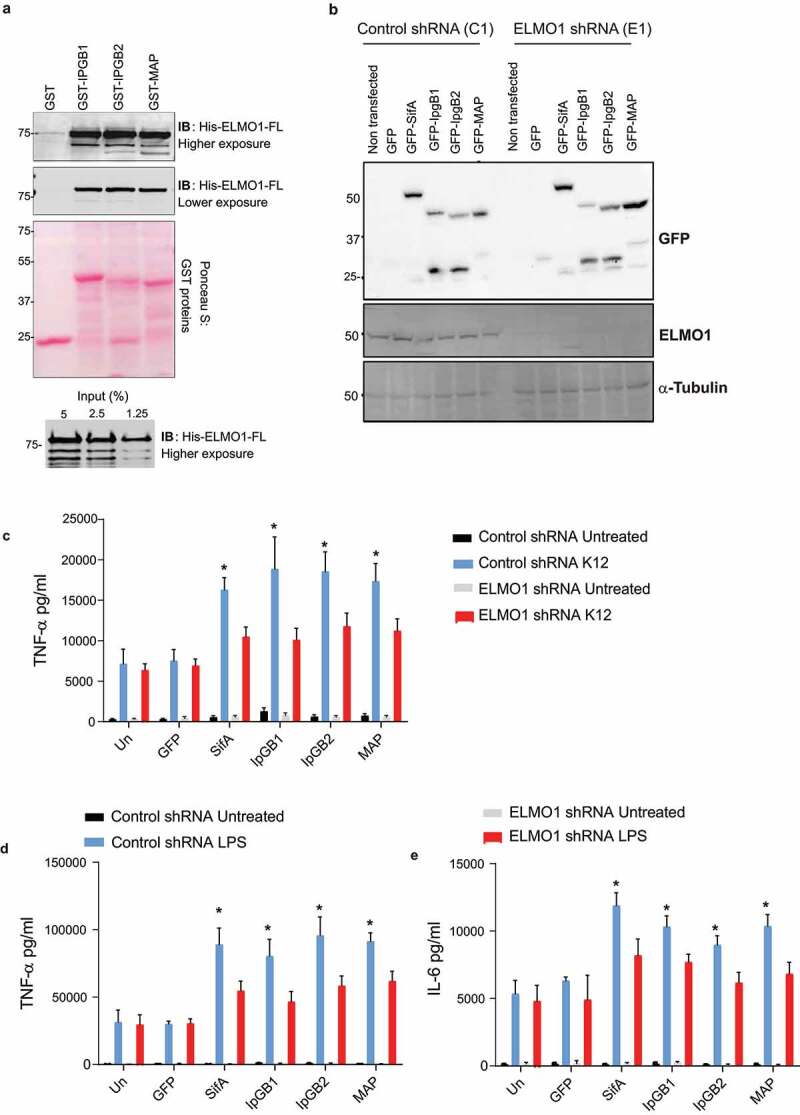
**Interaction of ELMO1 and bacterial effectors with WxxxE signature motif controls inflammatory responses** (a) (upper panel) Recombinant His-ELMO1-full length (FL) was incubated with GST, GST-IpgB1, GST-IpgB2, or GST-Map immobilized to glutathione sepharoseaffinity beads. The bound ELMO1-FL was visualized by immunoblot . Equal loading of GST proteins was confirmed by Ponceau S staining . (lower panel) The input of His ELMO-1- FL was shown. (b) Control (C1) or ELMO1 (E1) shRNA J774 cells were transfected with GFP vector control, GFP-SifA, GFP-IpgB1, GFP-IpgB2, and GFP-Map. The cell lysates were assessed by anti-GFP antibody (upper panel), and anti-ELMO1 antibody (middle panel). Equal loading was confirmed by α-Tubulin (lower panel). (c-e) Control or ELMO1 shRNA J774 cells were transfected with GFP vector, GFP-SifA, GFP-IpgB1, GFP -IpgB2, and GFP-Map as in (B), and then the cells were challenged with *E.coli* K12 (c) or LPS (d-e). The supernatants were collected and the level of TNF-α (c, d) and IL-6 (e) was measured by ELISA. Data in C-E represent mean ± SEM of three independent experiments. * means p ≤ 0.05 as assessed by one-way ANOVA multiple comparisons

To assess if ELMO1 interacts with WxxxE motif-containing effectors from *Shigella* (IpgB1, IpgB2) and *E. coli* (Map), we performed a GST pull-down assay using GST-IpgB1, GST-IpgB2, and GST-MAP with purified His-ELMO1 full length (FL) (Figure 5(a)). We found that ELMO1 interacts with all these bacterial effectors (Figure 5(a)). Next, we assessed the effect of overexpression of these effectors in control (C1) and ELMO1 (E1) shRNA J774 cells upon stimulation with bacteria and/or bacterial products such as LPS. To this end, control and ELMO1 shRNA cells were transfected with GFP-tagged bacterial effectors (SifA, IpgB1, IpgB2, and MAP). The overexpression of the bacterial effectors was confirmed by WB, and it was comparable in control and ELMO1-depleted cells and the interaction of bacterial effectors with endogenous ELMO1 was specific as there was no expression of ELMO1 in ELMO1 shRNA cells (Figure 5(b)). To assess the relevance of the ELMO1-bacterial effectors interaction on the immune response, we infected some of the transfected cells from Figure 5(b) with *E. coli* K12, a nonpathogenic bacterium that lacks the WxxxE motif, and we measured TNF-α levels in the supernatant. The level of TNF-α was increased with infection, and it was significantly higher in control (C1) cells transfected with SifA, IpGB1, IpGB2, and/or MAP compared to ELMO1 (E1) shRNA cells (Figure 5c). In contrast, the level of TNF-α was comparable in control and ELMO1 shRNA cells transfected with GFP empty vector (Figure 5(c)). Next, we stimulated with LPS both control and ELMO1 shRNA cells transfected with the bacterial effectors, and we measured TNF-α and IL-6 in the supernatant. Similarly, the level of TNF-α and IL-6 was increased following the LPS challenge, and the level of these cytokines was significantly higher in control (C1) cells transfected with bacteria effectors compared to transfected ELMO1 (E1) shRNA cells (Figures 5(d)**-5E)**.

## Discussion

The recent development of omics technology and the progress in the fields of microbiology and cell biology established the importance of physiological homeostasis in the intestinal mucosa. To maintain homeostasis, host immune signaling pathways need to discriminate between commensal and pathogens that cause infections. So far, we know partly about bacterial evasion mechanisms; how bacterial pathogens have evolved to avoid host immune defenses; and how pathogenic effectors downregulate inflammation to enable pathogens to reside within the host and avoid clearance.^27–30^ However, our knowledge is still limited on how host immunity discriminates between commensal and pathogenic microbes, balances the inflammatory cytokine responses, and helps the host to clear invading pathogens. Here, we have addressed how a host signaling pathway interacts with a subset of bacterial effectors, and how this interaction leads to inflammation, which may be important in controlling infections. Specifically, we have shown the interaction between the microbial sensor ELMO1 and a group of bacterial effectors that share a signature motif [WxxxE (Trp-x-x-x-Glu)]. Using *Salmonella* WxxxE effector SifA, we further investigated the impact of effector–host interactions in the ELMO1 KO mice model and in ELMO1-depleted macrophages.

Alto and colleagues described a large family of 24 WxxxE effectors present in enteric pathogens that mimic GTPases and use the downstream signaling of RhoA, Rac1, and Cdc42 without the need for GTP.^17^ The bacterial effectors include SifA, SifB of *Salmonella*; IpgB1, IpgB2 of *Shigella*; Map, EspT, and EspM of *E. coli*, which share the unique WxxxE motif (Figure 1). The BLAST search revealed that the WxxxE motif is widely distributed among enteric, non-enteric and plant pathogens but is absent in commensals (Figure 1, Supplementary Figures 1(a-d)). Interestingly, the TIR domain of human TLR proteins also has the WxxxE motif. Two type III effector proteins in plant pathogens, WtsE and AvrE, require the WxxxE motif for disturbing host pathways by mimicking activated host G-proteins.^31^ In enteric pathogens, WxxxE effectors functionally mimic the Rho family GTPase,^17,32^ and effectors such as IpgB1, IpgB2, and Map are mostly involved in the rearrangements of host cytoskeletal processes, probably to facilitate bacterial entry.^17,22^ The *Brucella* effectors BtpA or BtpB with the TIR domain have the WxxxE motif and are involved in protection against microtubule depolymerization.^33^ It is not known whether all WxxxE effectors share a similar function or play different roles depending on the pathogen. Our work will provide new insight into this functional aspect of pathogenesis.

The *Salmonella* effector SifA is secreted by the type-III secretion system encoded on the *Salmonella* pathogenicity island 2 (SPI-2). It is essential for the formation of the *Salmonella*-containing vacuole (SCV) and for extending the Sif membrane networks and the survival of the bacteria inside macrophages.^34–36^ SifA has the WxxxE motif,^17,35^ and its C-terminus is essential for maintaining the tertiary structure of SifA, which is crucial for the interaction with the SKIP protein important for microtubule formation.^23^ Furthermore, the W197 and E201 residues of the WxxxE are required for the binding of SifA with SKIP leading to the antagonism of G-protein Rab9.^37^ We found that the WxxxE motif is also important for the interaction between SifA and ELMO1. Since ELMO1 interacts with bacterial effectors containing WxxxE, these effectors can modulate host immune signaling to produce the appropriate response. Our results reveal that ELMO1 plays a role in the internalization of both pathogenic and nonpathogenic bacteria but stimulates the release of pro-inflammatory cytokines (e.g., TNF-α) only in response to pathogenic bacteria. Collectively, these findings indicate that ELMO1-WxxxE effector interaction could explain the differential immune response mediated by ELMO1 to discriminate between pathogens and commensals.

ELMO1 facilitates intracellular bacterial sensing and the induction of inflammatory responses following enteric infection.^9–11,16^ To gain additional insights, we evaluated the relevance of ELMO1–SifA interaction on bacterial internalization, pathogenesis, and host immune responses. Using WT and ELMO1 KO mice (global KO and myeloid cell-specific ELMO1 KO) challenged with WT *Salmonella* or the *sifA* mutant, we found that the infection was less severe in ELMO1 KO mice, particularly in mice infected with the *sifA* mutant compared to WT *Salmonella*. Our findings suggest that ELMO1–SifA interaction increases bacterial colonization, dissemination, histopathology score, and inflammatory response *in vivo*. It is possible that the caspase-3 cleavage of SifA may be required for bacterial dissemination^38^ and that there may be a link with ELMO1, but this needs to be further investigated. Previous work by us showed that ELMO1 is required for maximal bacterial internalization and pro-inflammatory responses during enteric infection.^9^ Here, we have further dissected the function of ELMO1 and showed the importance of specific bacterial effectors. It is already known that deletion of SifA impairs the ability of *Salmonella* to invade and replicate within the host cells, evade the host immune system, and disseminate to extraintestinal organs. Our study also correlates with the results from Patel *et al*.^38^ where they have reported that deletion of the SifA from *Salmonella* results in a 1-log lower dissemination of *Salmonella* to the liver. Therefore, the degree of infection and inflammation was decreased in mice infected with the *sifA* mutant compared to mice infected with WT *Salmonella*. Since ELMO1 is crucial for bacterial internalization and the induction of inflammatory response, infection with WT *Salmonella* was more attenuated in ELMO1 KO mice. ELMO1-depleted phagocytes exhibited significantly lower bacterial clearance, which also correlated to lower activity of lysosomal enzymes compared to control phagocytes.^10^ In addition, SifA stimulates host signaling analogous to the Rab GTPases, which is crucial to regulate membrane trafficking to the intracellular vacuole housing *Salmonella* in the host cytoplasm.^34,39,40^ Future cell biology and structural biology studies are required to understand the detailed downstream effects of ELMO1–SifA interaction in the maintenance of *Salmonella* vacuole and any endo-lysosomal machineries that control bacterial clearance.

Our study has shown the interaction between ELMO1 and bacterial effectors containing the WxxxE motif, and how this interaction is important for the generation of inflammation. We evaluated the impact of the interaction between ELMO1 and the WxxxE containing bacterial effectors on the inflammatory response released from macrophages *in vitro*. We found that the production of pro-inflammatory cytokines such as TNF-α and IL-6 was lowered in ELMO1-depleted macrophages with significantly lower amount in cells infected with the *sifA* mutant compared to cells infected with WT *Salmonella*. Likewise, lower cytokine levels were released from ELMO1-depleted macrophage transfected with other WxxxE containing effectors such as IpgB1, IpgB2, and Map upon stimulation with bacteria or bacterial products (LPS). The level of inflammatory transcripts was low in ELMO1 KO mice and in mice infected with the the *sifA* mutant. In a parallel line, previous reports showed that the *sifA* mutant cannot replicate and survive in macrophages and, therefore, cannot stimulate an inflammatory response.^41,42^ The common function of bacterial effectors is to down-regulate the immune signaling to hide inside the host. Here, we have shown how ELMO1 interacts with SifA and other WxxxE effectors and activates inflammatory pathways to alert the host about pathogens. Prior studies have shown that the EPEC effectors EspT and Map activate NFĸB and MAP kinase pathways to activate the immune signaling.^43,44^ We have shown that ELMO1 activates NFĸB and MAP kinase pathways following *Salmonella* infections.^9^ Future study is ongoing to understand the ELMO1–WxxxE effectors interaction that controls NFĸB/MAPK-mediated inflammatory signals that can trigger the production of cytokines.

In conclusion, our results in *Salmonella* have shown that ELMO1–SifA interaction promotes bacterial colonization, dissemination, and inflammatory immune response. We have provided evidence that ELMO1 can discriminate between enteric pathogens and commensal probably through interaction with the WxxxE containing effectors. The interaction between host engulfment protein ELMO1 and bacterial effectors is crucial to control the disease pathogenesis.

## Materials and methods

All methods involving animal subjects were performed in accordance with the relevant guidelines and regulations of the University of California San Diego and the NIH research guidelines.

### Bacteria and bacterial culture

*Salmonella enterica* serovar Typhimurium strain SL1344, *E. coli* strain K12 were obtained from the American Type Culture Collection (ATCC) (Manassas, VA, USA), *E. coli* DH5α was obtained from ThermoFischer Scientific, *E. coli* strain Nissle 1917 was obtained from Ardeypharm, and the SL1344 *sifA* mutant strain was obtained from Dr Olivia Steele-Mortimer.^36,45^ All bacteria were maintained as described previously.^9,46^ Briefly, a single colony was inoculated into LB broth and grown for 8 h under aerobic conditions and then under oxygen-limiting conditions. For the SL1344 *sifA* mutant, streptomycin with a final conc of 100 μg/mL was added to LB broth. Cells were infected with a multiplicity of infection (moi) of 10.

### Cell culture and transfection

HEK293 cells and murine macrophage cell line J774 were obtained from the American Type Culture Collection (Manassas, VA, USA). Cells were maintained in high glucose DMEM (Life Technologies) containing 10% fetal bovine serum and 100 U/ml penicillin and streptomycin at 37°C in a 5% CO_2_ incubator. Control (C1) and ELMO1 depleted (E1) shRNA J774 cells were generated as previously described^9^ and maintained in complete media supplemented with 0.5 μg/ml Puromycin (Sigma). Cells were sub-cultured 24 hours prior to transfection. Transfections of plasmids were performed using Lipofectamine 2000 (Invitrogen) according to manufacturer’s protocol.

#### Isolation of bone marrow-derived macrophages (BMDM) from mice

BMDM has been isolated using our previously published work.^9,12^ C57B6 mice (n = 7) aged 8–12 weeks were euthanized, and the femur bones were isolated. The ends of both femurs were cut, and the bone marrow cells were flushed using 25 G needle and RPMI media. The cell pellets were then centrifuged and incubated with 1X RBCs lysis buffer (Thermo Fisher Scientific) for 3 minutes to lyse the RBCs. The remaining bone marrow cells were precipitated and resuspended in DMEM media containing 10% FBS, 20% LCCM (L929 cells conditioned media), and ciprofloxacin (10 µg/ml) and incubated at 37°C. The media was changed after 3 days, and new media was added without the antibiotic. After 5–6 days, the cells were collected, lysed and used for pull down assay as described later.

### Blast search and sequence alignment

Literature was searched to find published research regarding WxxxE motifs in plants, enteric, and non-enteric pathogens. Protein and bioinformatics databases UniProt (https://www.uniprot.org/), PATRIC (https://www.patricbrc.org/), and NCBI’s BLAST (https://blast.ncbi.nlm.nih.gov/Blast.cgi) was used to search for protein sequences and BLAST for related proteins that may have the WxxxE motif. Clustal Omega (https://www.ebi.ac.uk/Tools/msa/clustalo/) was used to align sequences and Adobe InDesign CS6 was used to design the final figures that combined all effectors from each category (enteric pathogens, non-enteric pathogens, plant pathogens, and human Toll-Like receptors).

### *Infection of WT and ELMO1 KO mice with* Salmonella *strains*

To assess the role of ELMO1 and of SifA effector protein on bacterial pathogenesis *in vivo*, age- and sex-matched WT and ELMO1 KO (either global KO or LysM-cre driven where the ELMO KO was specifically deleted in myeloid cells) C57BL/6 mice were infected with *Salmonella enterica* serovar Typhimurium SL1344 wild-type or an isogenic *sifA* mutant (5 × 10^7^ cfu/mouse) by oral gavage. The infection period was 5 days, and the mice were monitored for weight change and clinical signs of disease during this period. At day 5 postinfection, the mice were sacrificed, and their tissues (liver, spleen, ileum, and cecum) were collected to assess bacterial colonization, inflammatory responses, and histology score. To assess the early kinetics of infection, WT and ELMO1 KO were challenged with SL1344 or the *sifA* mutant by oral gavage and the infection lasted for 2 days. Animals were bred, housed, used for all the experiments, and euthanized according to the University of California San Diego Institutional Animal Care and Use Committee (IACUC) policies under the animal protocol number S18086. All methods were carried out in accordance with relevant guidelines and regulations and the experimental protocols were approved by institutional policies and reviewed by the licensing committee.

### Assessment of bacterial load and inflammatory response in the mice tissues

Harvested tissues were weighed first and then resuspended in phosphate-buffered saline (PBS), homogenized, diluted serially, and plated in LB agar plates. The *sifA* mutant was plated in LB agar plates with Streptomycin (Sigma, final conc 100 μg/mL). The bacterial load was calculated as colony forming unit (cfu) per gram of tissue. To assess the expression of inflammatory cytokines, tissue specimens from spleen, ileum, cecum, and liver were collected for RNA isolation from these tissues.

### RNA preparation, real-time reverse-transcription polymerase chain reaction

Total RNA was extracted using Direct-zol RNA MiniPrep Kit (Zymo Research, USA) according to the manufacturer’s instructions. cDNA was synthesized using the qScript™ cDNA SuperMix (Quantabio). Quantitative PCR (qPCR) was carried out using 2x SYBR Green qPCR Master Mix (Biotool™, USA) for target genes that normalized to the endogenous control gene using the 2^−ΔΔCt^ method. Primers were designed using NCBI Primer Blast software and the Roche Universal Probe Library Assay Design software (Table 1).

**Table:** 

Gene	Forward Primer (5ʹ →3ʹ)	Reverse Primer (5ʹ →3ʹ)
**18srRNA**	GTAACCCGTTGAACCCCATT	CCATCCAATCGGTAGTAGCG
**β-actin**	GACGGCCAGGTCATCACTAT	ACATCTGCTGGAAGGTGGAC
**TNF-α**	CCACCACGCTCTTCTGTCTA	AGGGTCTGGGCCATAGAACT
**IL-1β**	GAAATGCCACCTTTTGACAGT	CTGGATGCTCTCATCAGGACA
**MCP-1**	AAGTGCAGAGAGCCAGACG	TCAGTGAGAGTTGGCTGGTG
**IL-6**	CCCCAATTTCCAATGCTCTCC	CGCACTAGGTTTGCCGAGTA
**CXCL-1**	CGCTTCTCTGTGCAGCGCTGCTGCT	AAGCCTCGCGACCATTCTTGAGTC

### *Histopathology of WT and ELMO-1 KO mice following* Salmonella *infection*

Ileal tissues from SL1344 and *sifA* mutant-infected WT and ELMO-1 KO mice were fixed using Zinc formalin and stained with H&E. The slides were evaluated for the presence of inflammatory cells, such as neutrophils, mononuclear infiltrates, and mucosa architecture architecture, and the histology score for each slide was determined as described previously.^9,16^

### *Infection of J774 macrophages with* Salmonella *strains and nonpathogenic bacteria for cytokine assays*

Control (C1) and ELMO1 (E1) depleted J774 macrophages cells were either left uninfected or infected with *Salmonella* strains (SL1344, and an isogenic *sifA* mutant), or nonpathogenic bacteria (*E. coli* strains K12, DH5α, and Nissle 1917 (Nissle A Über die Grundlagen einer neuen ursächlichen Bekämpfung der pathologischen Darmflora Deut Med Wochenschr 1916 42 1181 4)). Supernatants were collected from the infected cells and tested for TNF-α and IL-6, using a mouse ELISA kit (R&D Systems, USA) according to the manufacturer’s instructions.

### The impact of bacterial effectors on cytokine responses in J774 macrophages

Control (C1) and ELMO1-depleted (E1) J774 macrophage cells were transfected with plasmids containing the bacterial effectors (SifA, IpgB1, IpgB2, and Map) as described in the previous section. Transfected cells were challenged with LPS (100 ng/ml) for 6 h, and *E. coli* K12 (moi 10) for 3 h. Supernatants were collected at the time points and assessed for cytokines by ELISA.

### Bacterial internalization by gentamicin protection assay

Approximately 2 × 10^5^ cells were seeded into 24-well culture dishes 18 hours before the infection and infected with bacteria with moi 10 for 1 hours in antibiotic-free media as described previously.^9,11^

### Protein expression and purification

Recombinant His and GST-tagged proteins were expressed in *the Escherichia coli* strain BL21 (DE3) and purified as previously described.^47,48^ Briefly, proteins in bacterial culture were induced with IPTG (0.5 mM) overnight incubation at 25°C. Bacteria culture was centrifuged and cell pellet was lysed in either GST lysis buffer (25 mM Tris-HCl (pH 7.4) 20 mM NaCl, 1 mM EDTA, 20% (v/v) glycerol, 1% (v/v) Triton X-100, protease inhibitor cocktail) or His lysis buffer (50 mM NaH_2_PO_4_ (pH 7.4), 300 mM NaCl, 10 mM imidazole, 1% (v/v) Triton-X-100, protease inhibitor cocktail). Cell lysates were briefly sonicated and then centrifuged at 4°C for 30 mins at 12,000xg. Cleared cell lysates were then affinity purified using either glutathione-Sepharose 4B beads or HisPur Cobalt Resin, followed by elution and overnight dialysis in PBS. Proteins were then quantified and stored at −80°C.

### In vitro *GST pulldown and immunoprecipitation*

Recombinant purified GST-tagged proteins were immobilized onto the glutathione-Sepharose beads in a binding buffer (50 mM Tris-HCl (pH 7.4), 100 mM NaCl, 0.4% (v/v) Nonidet *P*-40, 10 mM MgCl2, 5 mM EDTA, 2 mM DTT) for 2 hrs at 4°C with gentle agitation. GST-tagged protein-bound beads were washed and incubated with purified His-tagged proteins or cell lysate in a binding buffer overnight at 4°C with gentle agitation. The GST beads were then washed four times with NETN buffer (0.5% NP40, 0.1 mM EDTA, 20 mM Tris, pH 7.4, 300 mM NaCl) for cell lysates or GST wash buffer (20 mM Tris, pH 7.4, 0.1 mM EDTA and 100 mM NaCl) for purified proteins.

For immunoprecipitation, transfected HEK293 cells or murine macrophage cell line J774 and bone marrow-derived macrophages (BMDM) were lysed in RIPA lysis buffer (50 mM Tris pH 7.4, 150 mM NaCl, 0.1% SDS, 1% NP40, 0.5% Sodium Deoxycholate) with 1X Proteinase Inhibitor Cocktail added immediately before use. Whole-cell lysate was centrifuged to separate proteins from cell debris and quantified using the Lowry assay. One mg of total protein lysate was diluted 4 times its volume with cold 50 mM Tris pH 7.4 (1X Proteinase Inhibitor Cocktail added fresh) and incubated with 40ul of antibody-conjugated beads overnight at 4°C with gentle agitation. Beads were washed 4 times with cold NP40 buffer (1% NP40, 0.5% Sodium Deoxycholate and 0.1% SDS in 1 X PBS) at 1500 rpm for 2 mins.

Bound proteins were eluted by boiling beads for 10 mins at 95°C in 2X SDS-PAGE sample buffer containing β-mercaptoethanol. Proteins were separated using SDS-PAGE protein gel and transferred to Immobilon-P PVDF membrane. Proteins were visualized by immunoblotting with corresponding antibodies.

### Assessment of Rac1 activity in control and ELMO1 shRNA cells

The activity of Rac1 was assessed by using GST-PBD (glutathione S-transferase with p21-binding domain of Pak1) beads as described before.^11^ Briefly, control and ELMO1 shRNA cells infected cells with WT SL or *sifA* mutant were lysed in buffer containing 50 mM Tris-HCl (pH 7.5), 2 mM MgCl2, 0.1 M NaCl, 1% NP-40 and 10% glycerol with protease inhibitors and incubated with GST coupled to PBD to precipitate Rac-GTP. The blots were visualized by electrochemiluminescence reagents (Pierce SuperSignal). The level of active Rac1 (GTP bound) was normalized to total Rac1 using ImageJ software.

### Statistical analysis

Results presented in this study were presented as the mean ± SEM and the bacteria load (CFU) was expressed as the geometric mean. Data compared using Mann–Whitney U-test, two-tailed Student’s t-test, and/or one-way ANOVA multiple comparisons as described in the specific positions. The results were analyzed in the Graph Pad Prism and considered significant if *p* values were <0.05.

## Supplementary Material

Supplemental Material

## Data Availability

Data available within the article or its supplementary materials.

## Highlights

1 – ELMO1 generates a differential immune response between enteric pathogens and commensals.

2 – Enteric bacterial effectors containing WxxxE signature motif interact with ELMO1.

3 – The WxxxE effector of *Salmonella* SifA interacts with the C-terminal part of ELMO1.

4 – ELMO1–SifA interaction increases the inflammatory response *in vivo* and *in vitro*.
