# Supplementary material for: The interaction of enteric bacterial effectors with the host engulfment pathway control innate immune responses
Source: Gut Microbes. 2021 Nov 1;13(1):1991776. doi: 10.1080/19490976.2021.1991776 (PMC8565811; doi:10.1080/19490976.2021.1991776)
Supplement: Supplemental Material [file KGMI_A_1991776_SM2160.zip › Supplementary figure legends.docx]

**Supplementary Figures Legends**

**Figure 1S: The distribution of the WxxxE motif among enteric, non-enteric, and host receptor.**

A BLAST search between amino acid sequences showed effectors with the WxxxE or “Trp-x-x-x-Glu” sequences within enteric pathogens (A), non-enteric pathogen (B), plant pathogens (C) and host proteins (D). (A) represents the list of enteric pathogens includes the name of effector and bacteria (left), the sequence ID (middle), and the amino acid sequences of the effectors (right). In the amino acid sequences WxxxE sequence is highlighted, where W and E are yellow highlighted, and xxx sequence is green highlighted. (B) represents the list of non-enteric pathogens includes the name of effector and bacteria (left), the sequence ID (middle), and the amino acid sequences of the effectors (right). (C) represents the list of plant pathogens includes the name of effector and bacteria (left), the sequence ID (middle), and the amino acid sequences of the effectors (right). (D) represents the list of human TLR containing this motif including the sequence ID and the name of receptor (left), and the amino acid sequences of the effectors (right). (E) Bacterial cell lysate expressing GST-SifA was immobilized onto Glutathione-Sepharose beads. The beads were incubated with lysates from control and ELMO1 shRNA J774 cells, and BMDM cells for immunopulldown. The pulldown samples were immunoblotted with anti-ELMO1 antibody. The equal loading of beads were confirmed by staining with Ponceau.

**Figure 2S:** **Effect of WT *SL* and *sifA* mutant infections on the bacterial colonization in Lys M-cre-driven ELMO1 KO mice.**

WT and myeloid cells specific ELMO1 KO mice (Lys M-cre) were infected via oral gavage with WT *SL* and *sifA* mutant strain for 5 days. (A-B) Bacterial burden was assessed at day 5 of infection in the ileum (A), and spleen (B) of the infected mice. *, ** mean *p* ≤ 0.05, and ≤ 0.01, respectively as assessed by Mann Whitney test.

**Figure 3S:** **Effect of WT *SL* and *sifA* mutant infections for 2 days on the bacterial colonization in global ELMO1 KO mice.**

WT and global ELMO1 KO mice were infected via oral gavage with WT *SL* and *sifA* mutant strain for 2 days. (A) The percentage of weight loss was assessed in WT and global ELMO1 KO mice after 2 days of infection. (B-E) Bacterial burden was assessed at day 2 of infection in the cecum (B), spleen (C), Liver (D), and ileum (E) of the infected mice. *, ** mean *p* ≤ 0.05, and ≤ 0.01, respectively as assessed by Mann Whitney test.

**Figure 4S: Effect of WT *SL* and *sifA* mutant infections for 2 days on the inflammatory cytokines in global ELMO1 KO mice.**

WT and global ELMO1 KO mice were infected via oral gavage with WT *SL* and *sifA* mutant strain for 2 days. Total RNA was isolated from the spleen and ileum of WT and global ELMO1 KO mice. (A-E) The transcript level of inflammatory cytokines: TNF-α (A), IL-6 (B), MCP-1 (C), IL-β (D), and CXCLl-1 (E) was measured by RT-qPCR in the spleen of infected mice. (F-J) The transcript level of inflammatory cytokines: TNF-α (F), MCP-1 (G), IL-6 (H), IL-1β (I), and CXCLl-1 (J) was assessed by RT-qPCR in the ileum of infected mice.
