# Supplementary figures and images for: The interaction of enteric bacterial effectors with the host engulfment pathway control innate immune responses
Source: Gut Microbes. 2021 Nov 1;13(1):1991776. doi: 10.1080/19490976.2021.1991776 (PMC8565811; doi:10.1080/19490976.2021.1991776)

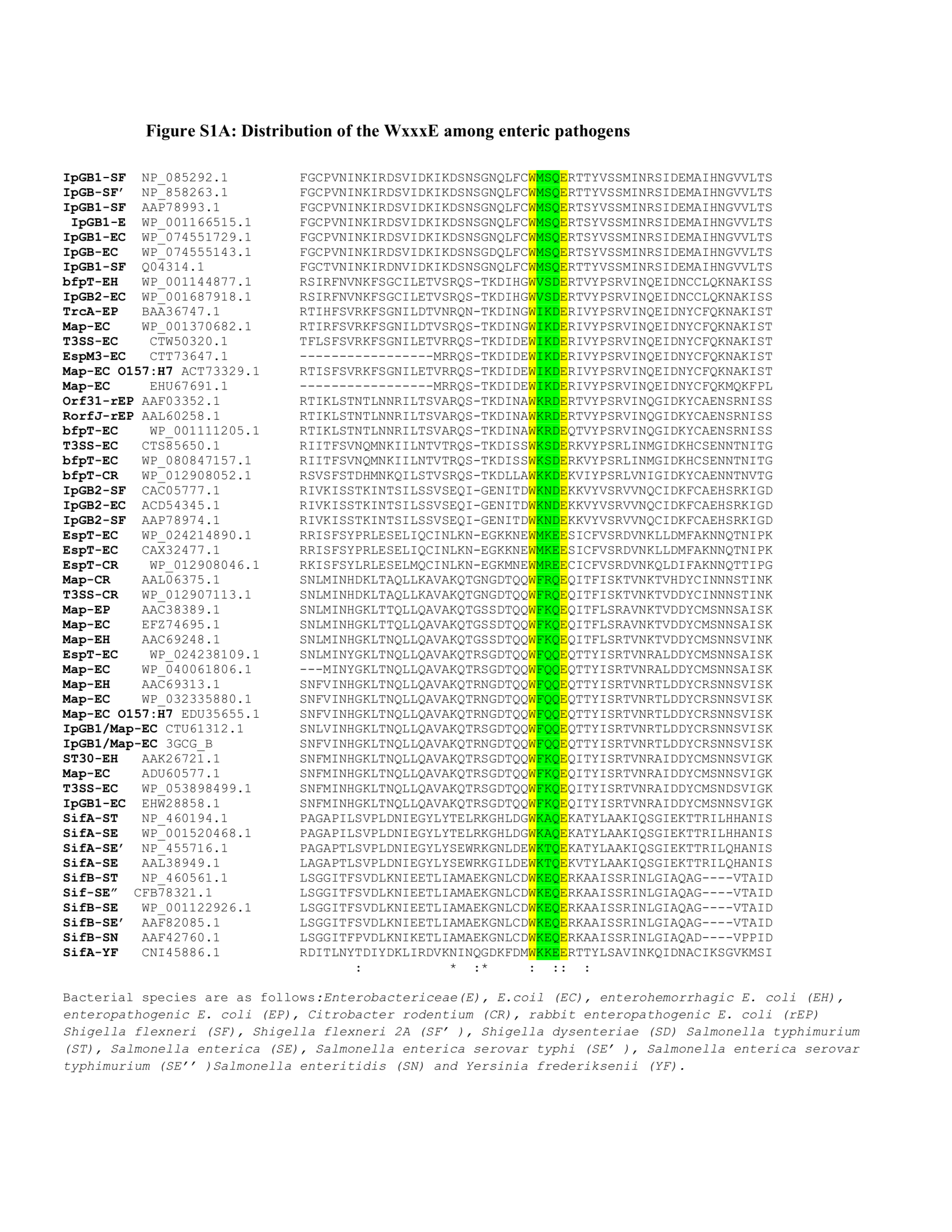

Supplement: Supplemental Material [file KGMI_A_1991776_SM2160.zip › Supplementary Figure S1A.tif]

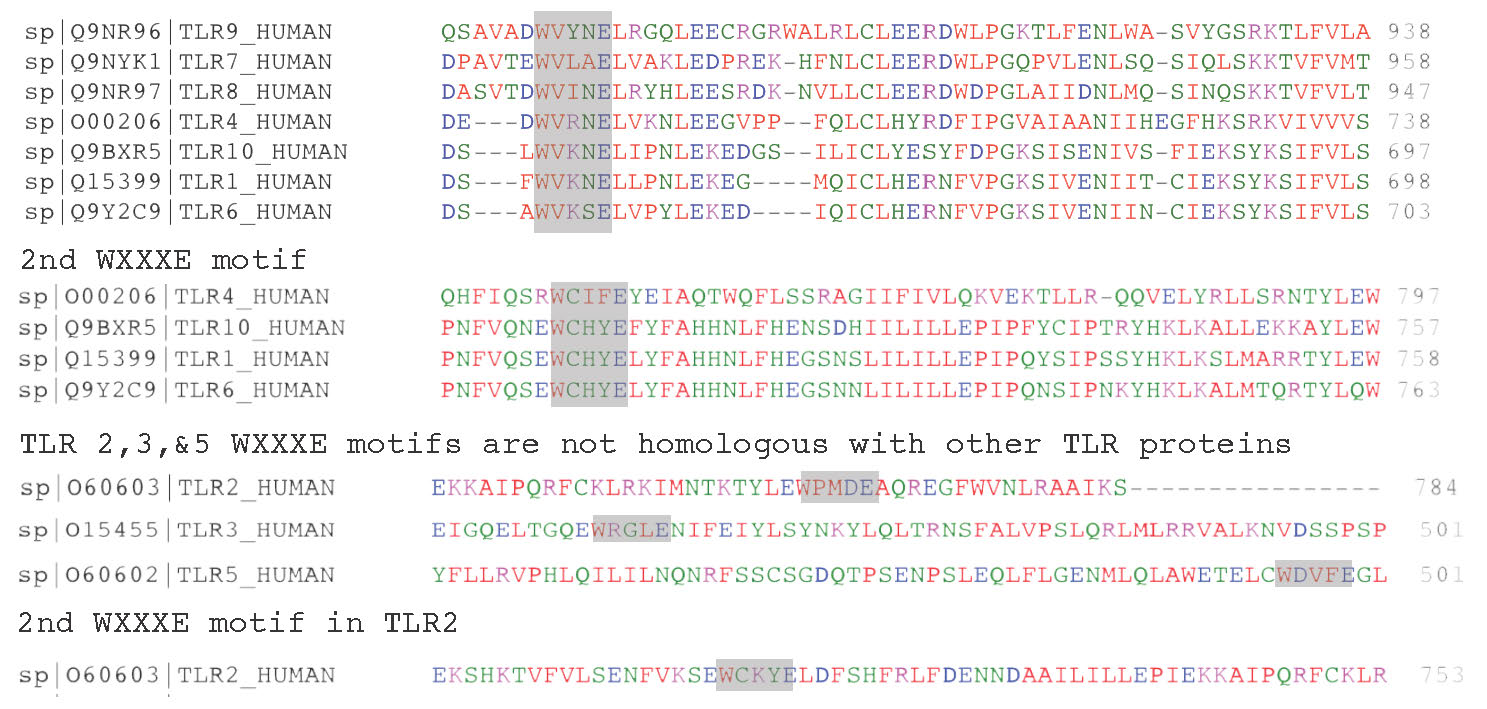

Supplement: Supplemental Material [file KGMI_A_1991776_SM2160.zip › Supplementary Figure S1D.jpg]

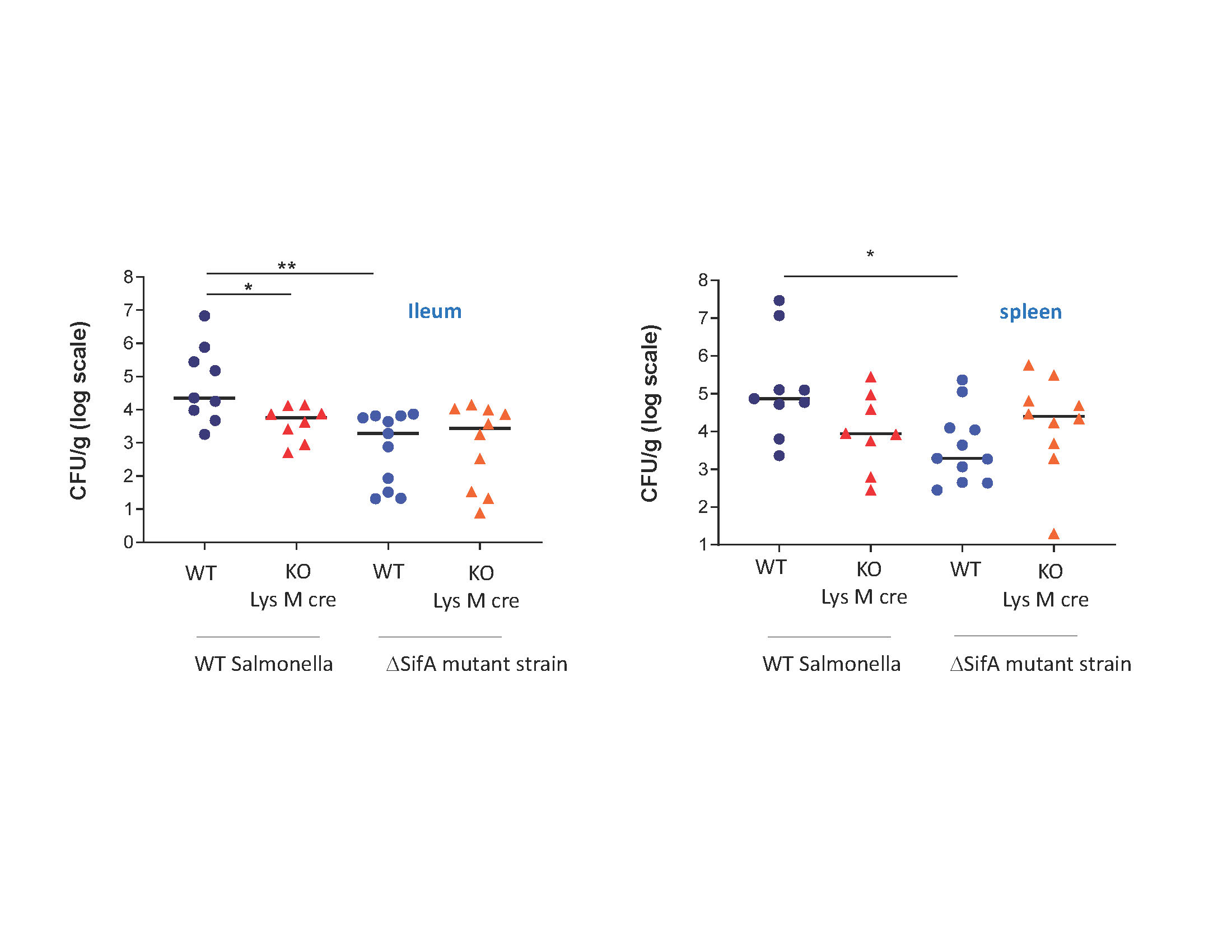

Supplement: Supplemental Material [file KGMI_A_1991776_SM2160.zip › Supplementary Figure S2.jpg]
